# Unveiling the genetic architecture for lodging resistance in rice (*Oryza sativa*. L) by genome-wide association analyses

**DOI:** 10.3389/fgene.2022.960007

**Published:** 2022-09-06

**Authors:** Muhammad Abdul Rehman Rashid, Yong Zhao, Farrukh Azeem, Yan Zhao, Hafiz Ghulam Muhu-Din Ahmed, Rana Muhammad Atif, Yinghua Pan, Xiaoyang Zhu, Yuntao Liang, Hongliang Zhang, Danting Li, Zhanying Zhang, Zichao Li

**Affiliations:** ^1^ State Key Laboratory of Agrobiotechnology/Beijing Key Laboratory of Crop Genetic Improvement, College of Agronomy and Biotechnology, China Agricultural University, Beijing, China; ^2^ Department of Bioinformatics and Biotechnology, Government College University Faisalabad, Faisalabad, Pakistan; ^3^ College of Agronomy, Shandong Agricultural University, Tai’an, China; ^4^ Department of Plant Breeding and Genetics, Islamia University Bahawalpur, Bahawalpur, Pakistan; ^5^ Department of Plant Breeding and Genetics, University of Agriculture Faisalabad, Faisalabad, Pakistan; ^6^ Precision Agriculture and Analytics Lab, National Centre in Big Data and Cloud Computing, Centre for Advanced Studies in Agriculture and Food Security, University of Agriculture Faisalabad, Faisalabad, Pakistan; ^7^ Rice Research Institute, Guangxi Academy of Agricultural Sciences/Guangxi Key Laboratory of Rice Genetics and Breeding, Nanning, China

**Keywords:** GWAS, association mapping, rice (*Oryza sativa* L.), lodging resistance, culm strength, genetic architecture

## Abstract

Lodging is one of the major abiotic stresses, affecting the total crop yield and quality. The improved lodging resistance and its component traits potentially reduce the yield losses. The section modulus (SM), bending moment at breaking (M), pushing resistance (PR), and coefficient of lodging resistance (cLr) are the key elements to estimate the lodging resistance. Understanding the genetic architecture of lodging resistance–related traits will help to improve the culm strength and overall yield potential. In this study, a natural population of 795 globally diverse genotypes was further divided into two (*indica* and *japonica*) subpopulations and was used to evaluate the lodging resistance and culm strength–related traits. Significant diversity was observed among the studied traits. We carried out the genome-wide association evaluation of four lodging resistance traits with 3.3 million deep resolution single-nucleotide polymorphic (SNP) markers. The general linear model (GLM) and compressed mixed linear model (MLM) were used for the whole population and two subpopulation genome-wide association studies (GWAS), and a 1000-time permutation test was performed to remove the false positives. A total of 375 nonredundant QTLs were observed for four culm strength traits on 12 chromosomes of the rice genome. Then, 33 pleiotropic loci governing more than one trait were mined. A total of 4031 annotated genes were detected within the candidate genomic region of 33 pleiotropic loci. The functional annotations and metabolic pathway enrichment analysis showed cellular localization and transmembrane transport as the top gene ontological terms. The *in silico* and *in vitro* expression analyses were conducted to validate the three candidate genes in a pleiotropic QTL on chromosome 7. It validated *OsFBA2* as a candidate gene to contribute to lodging resistance in rice. The haplotype analysis for the candidate gene revealed a significant functional variation in the promoter region. Validation and introgression of alleles that are beneficial to induce culm strength may be used in rice breeding for lodging resistance.

## 1 Introduction

Rice (*Oryza sativa* L.), as one of the main staple food crops, is very important for food security worldwide (Meng et al., 2021). Among the various biotic and abiotic stresses, lodging is a major factor, causing significant reduction in grain yield and quality by decreased photosynthesis and nutrient transportation (Berry et al., 2004; Islam et al., 2007). The modern high yielding varieties bred for long spikes and high grain weight become vulnerable to stem lodging (Hirano et al., 2017). The field management with more fertilizer consumption and high planting density leads to reduced culm strength and an increased risk of lodging (Shah et al., 2019). Previously, reduced plant height with semi-dwarf gene *sd1* caused a green revolution and decreased the risk of lodging in cereals (Peng et al., 1999; Sasaki et al., 2002). However, the clear genetic architecture of lodging resistance is still unclear.

There are two main types of lodging in rice, that is, stem lodging and root lodging. The current study focused on the traits associated with lodging resistance of rice culm. The significant association of lodging resistance with culm strength has been reported in rice (Meng et al., 2021). Culm strength is one of the complex quantitative traits that are contributed by multiple traits, including culm morphology, diameter, thickness, plant height, and stem stress bearing capacity, due to accumulation of macromolecules like lignin and carbohydrates (Kashiwagi and Ishimaru, 2004; Kashiwagi et al., 2006; Yano et al., 2015; Fan et al., 2018; Sowadan et al., 2018). Culm lodging can be further classified as culm-breaking and culm-bending types. Both of the culm strength indicators often occur in transplanting cultivation. The culm-breaking can be estimated by the bending moment at breaking (M), which is the measure of culm thickness calculated as section modulus (SM) and culm stiffness calculated as bending stress (BS) (Ookawa et al., 2010; Chigira et al., 2020). Meanwhile, the culm flexibility can also be evaluated as the coefficient of lodging resistance (Grafius and Brown, 1954).

Some studies and breeding trials have been conducted to improve the culm strength, but they have not succeeded due to the negative correlation of culm strength (tallness and thickness) and yield parameters (Hirano et al., 2017). Exploitation of potential genetic resources, identification of novel loci governing the culm strength, and gene pyramiding are the promising solution to improve yield and strength simultaneously (Meng et al., 2021). Recently, various genes/QTLs governing lodging resistance and/or its contributing traits have been reported. The QTLs *SCM1* and *SCM2* were reported to induce culm strength. *SCM2*, similar to the *APO1* gene, controls the culm diameter and culm morphology by encoding an F-box domain containing protein (Ookawa et al., 2010; Rashid et al., 2016). A QTL *SCM3* was reported to control the culm morphology by affecting the strigolactone signaling pathway (Yano et al., 2015; Meng et al., 2021). The *gnla* mutant could improve the lodging resistance by increasing culm diameter (Tu et al., 2022). The COBRA protein encoding gene *BC1* was reported to contribute the culm strength by cell wall cellulase microfilaments elongation and cell wall thickening (Li et al., 2003). The *sd1* gene and TUT1 allele *es1-1* were reported for short stature, resulting in lodging resistance. In addition to these genes, the genetic studies on internode length, grain weight, panicle length, and tiller angle (Rashid et al., 2022) have also been observed to contribute to culm strength, leading to lodging resistance in rice.

In recent years, rice researchers widely used the genome-wide association studies (GWASs) to investigate the complex traits of agronomic and commercial importance including yield parameters, flowering time (Huang et al., 2010; Huang et al., 2012; Rashid et al., 2022), and abiotic stresses like drought (Al-Shugeairy et al., 2015) and salinity (Kumar et al., 2015). Nonetheless, a few GWAS studies were conducted for lodging resistance (Hu et al., 2013; Meng et al., 2021) in crops. In this research, a genome-wide association study (GWAS) was conducted for the SM, M, PR, and cLr. The regression models, such as general linear model (GLM), mixed linear model (MLM), and compressed mixed linear model (cMLM), were used to estimate the marker trait association (MTA). The 1000-times permutation test was also used to remove false-positives. Moreover, the combination of various regression models in GWAS helped to control false positives (Wu et al., 2016; Misra et al., 2017; Zhang et al., 2018). This study clearly aimed to explore the genomic regions associated with lodging resistance in the whole rice genome. We investigated the genetic variation and heritability among lodging resistance traits, their statistical and biological correlation, significantly associated loci, pleiotropic loci, and candidate genes. The research will not only provide a deep insight into the genetic basis of lodging resistance but also point toward improving yield potential in rice breeding programs.

## 2 Materials and methods

### 2.1 Plant material and field management

The study was conducted by using a natural population of 795 genetically and geographically diverse rice genotypes selected from a full population of 3,000 rice genome projects (3KRGP) (Li J. Y. et al., 2014; Li Z. et al., 2014). The germplasm was selected as a representative population including the rice mini-core collection from China Agricultural University (CAU) China and 525 diverse breeding lines from the Chinese Academy of Agricultural Sciences China as previously described (Zhao et al., 2018a; Zhao et al., 2018b; Rashid et al., 2022). The genetic purity and homozygosity of germplasm was confirmed by growing the plants through a single seed descent (SSD) approach for 3 years (2013–2016). The experimental plant material was grown in a randomized complete block design (RCBD) in three replications for 3 consecutive years. Two experimental blocks at the bird-net secured experimental farm of China Agricultural University, Sanya, Hainan (18 ^o^N, 109 ^o^E), China. Three lines of 1 m length for each genotype were grown, and seven seeds per line were maintained. Sampling and phenotyping were performed on three guarded plants from each line. The average value of three replicated plants from each block during 3 years (Yu et al., 2007) was used for analysis. The standard cultural practices as per local area requirements were kept constant.

### 2.2 Phenotyping

The lodging resistance of three guarded plants from each of two experimental blocks was calculated by the standard lodging resistance parameters, including section modulus (SM), pushing resistance (PR), bending moment at breaking (M), and coefficient of lodging resistance (cLr). SM was measured in cubic-millimeters (mm^3^) by the formula (Ookawa et al., 2010):

$$SM=\text{π}\cdot \frac{\left\{a_{1}^{3}b_{1}-a_{2}^{3}b_{2}\right\}}{32a_{1}},$$

where a_1_ is the culm outer diameter of the minor axis in an oval cross-section, b_1_ is the outer diameter of the major axis in an oval cross-section, a_2_ is the inner diameter of the minor axis in an oval cross-section, and b_2_ is the inner diameter of the major axis in an oval cross-section. The whole plant’s pushing resistance (PR), also known as bending stress at breaking (BnS), was measured in grams per centimeter-square (g/cm^2^) with a lodging meter at the maturity stage, according to a method reported previously (Kashiwagi and Ishimaru, 2004). The bending moment (M) of the basal internode at breaking (g/cm) was calculated as follows as previously described (Ookawa et al., 2016):

M=Section Modulus (SM)×Bending Stress (BnS).

The coefficient of lodging resistance (cLr) value of the plant was determined in g/cm according to the method described by Grafius and Brown (Grafius and Brown, 1954). The average value of ten individual plants was considered the final reading for the succeeding analysis.

### 2.3 Germplasm genotyping and population structure evaluation

The second generation of whole-genome high-throughput sequences with ∼13-fold coverage was obtained by Illumina sequencing, and the sequence reads were aligned against *japonica* cv. *Nipponbare* reference genome. The direct comparison with corresponding regions in the reference sequence produced 10 million raw SNPs. The SNP screening was performed with threshold criteria of missing rate more than 20 percent and at least 5% minor allele frequency. The completed set of selected SNP markers distributed over the 12 chromosomes of the rice genome were used to calculate the population structure parameters.

The population structure (Q-matrix), principal components (PCs) and kinship (K) matrix were calculated by the GAPIT program (http://www.maizegenetics.net/GAPIT). The 795 individuals in the full population could be divided into two sub-populations as *japonica* and *indica* based on PCs and K-matrix. Later on, the kinship matrix and the principal components were re-estimated for each sub-population to be used as covariates in the mixed linear model (MLM) of regression analysis. To map the known genes and candidate loci, their physical positions were acquired from the online available rice database (http://rapdb.dna.affrc.go.jp).

### 2.4 Statistical analysis of phenotypes

The statistical descriptive for phenotypic or morphological data was calculated by SPSS version 19 (http://www-01.ibm.com/software/analytics/spss/). The F-distribution test was performed to evaluate the significance of available diversity among genotypes. The analysis of variances (ANOVA) was performed to estimate the genotypic variance (V_g_) and environmental variance (V_e_). The interaction variances were estimated by using the STATISTICA software (StatSoft 1995; Tulsa, OK, United States). The experimental repeatability (*r* ^2^) and the broad-sense heritability (h^2^) (Sukumaran et al., 2015) were estimated by using genotypic variance (V_g_), error variance (V_e_), and interaction variances as previously described (Rashid et al., 2022). All the variance components were estimated by ANOVA in the F-distribution test. The frequency distribution–based data normality was confirmed by the Q-Q plot drawn by the SPSS program and GAPIT scripts in the R-program.

### 2.5 Genome-wide association analysis

The average value of ten plants for each genotype was used as the final estimate of each trait. For the marker trait association for lodging resistance related traits, two regression models, viz, general linear model (GLM) and compressed mixed linear model (cMLM) were applied to three sets of populations as (i) whole population, (ii) *indica* sub-populations, and (iii) *japonica* sub-populations. In the GLM model, the population structure (PCAs) was incorporated as a covariate. The population structure (Q-matrix) and kinship matrix (K) were included as fixed and random effects, respectively, to conduct a compressed mixed linear model (cMLM). The 1,000-permutation test by the mixed linear model MLM was performed for detection and screening of false SNP signals.

### 2.6 Identification of significant QTLs, candidate genes, and pleiotropic loci

The QTL regions were strictly defined by linkage disequilibrium (LD) blocks of 167 kb and 123 kb left and right genomic regions of significant SNPs (SNP ± LD) for *japonica* and *indica* subpopulations, respectively, and 170 kb for the whole population, as previously reported (Huang et al., 2010; Huang et al., 2012; Zhao et al., 2018a; Zhao et al., 2018b). To estimate a significance threshold level, we used the formula “−log10 (0.01/effective number of SNPs with a *p*-value less than 0.01),” that is, the threshold at a significance level of 1% after Bonferroni-adjusted multiple test correction (Zhao et al., 2018b). The QTLs with no annotated loci were removed. The significant QTLs were further screened by deletion of non-coding SNPs residing other than CDS or promoter regions of annotated loci. The significant loci identified for more than one lodging resistance parameter were considered pleiotropic loci.

### 2.7 Functional annotation and pathway enrichment analysis

All the candidate loci within the QTL range of pleiotropic loci were subjected to detect their functional annotation. The candidate genes list was submitted to ShinyGo 0.76 (Ge et al., 2019) (http://bioinformatics.sdstate.edu/go/) for gene ontological analysis and the same list was submitted to the Database for Annotation, Visualization, and Integrated Discovery (DAVID) (Sherman et al., 2022) for functional pathway evaluation based on the Kyoto Encyclopedia of Genes and Genomes (KEGG) (Kanehisa et al., 2020) pathway database.

### 2.8 *In silico* and *in vitro* expression estimation

The *in silico* expression profile of top candidate genes was further evaluated in the rice gene expression database (RED) (http://expression.ic4r.org/). Total RNA was isolated from the stem part collected at fourth internode of the strong and weak plants using the RNA-prep pure plant kit (TIANGEN) and reverse transcribed using HiScript II QRT SuperMix (Vazyme) (Chun et al., 2020). Quantitative real-time PCR for selected candidate genes in a pleiotropic QTL on chromosome 7 was performed using SYBR green mixture on an ABI 7500 real-time PCR detection system. Rice *Ubiquitin* gene was used as an internal control for normalization (Chun et al., 2020). All primer sequences are listed in Supplementary Table S1.

### 2.9 Haplotyping of candidate genes

The total gene length of candidate genes were obtained and SNPs in the promoter and coding gene region were extracted. All the significant and non-significant SNPs in the promoter and CDS regions of selected loci were used to evaluate their functional haplotypes (Rashid et al., 2016). The SNP markers were aligned and arranged in a Microsoft Excel sheet and saved as a text file in FASTA format. The program DnaSP was used for haplotype analysis, and the results were visualized by Prism 8 software (Librado and Rozas, 2009).

## 3 Results

### 3.1 Trait diversity and heritability among lodging resistance traits

**FIGURE 1:**
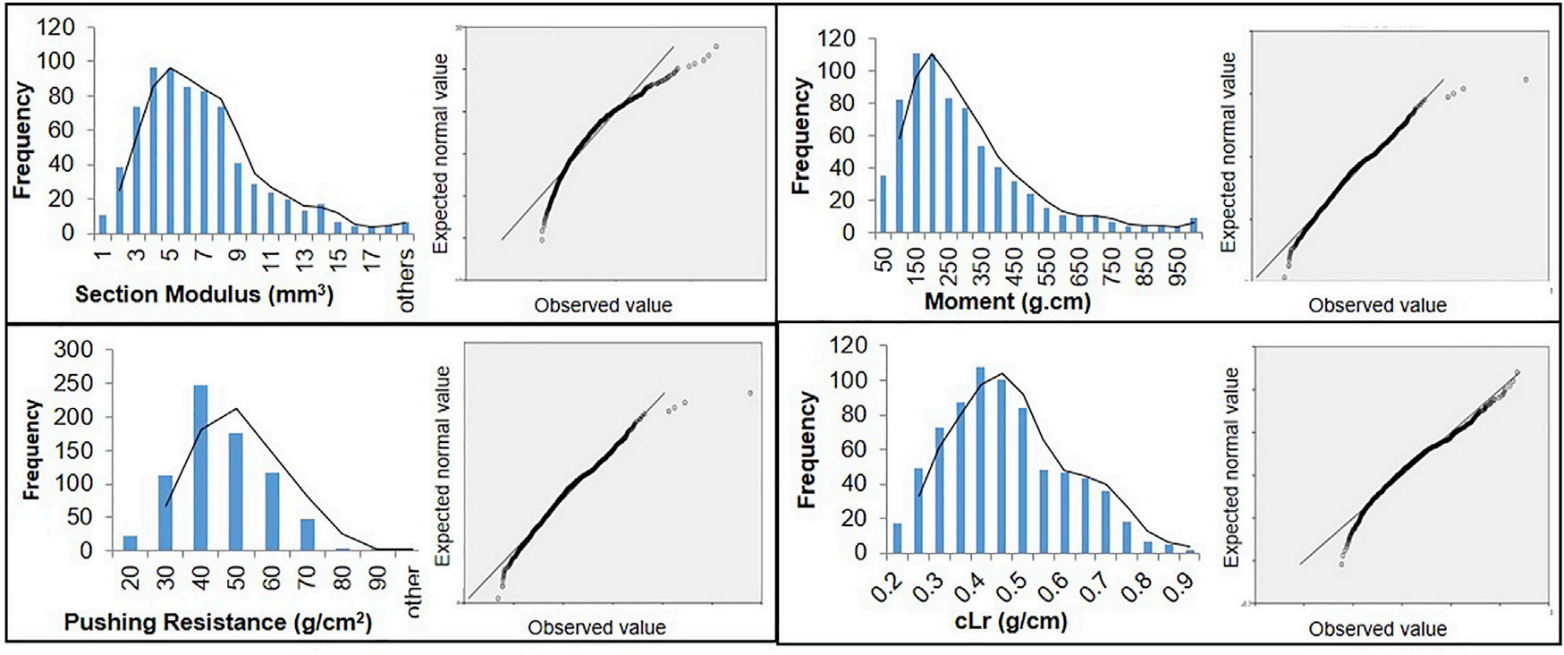
Frequency distribution and Q-Q plots of 795 genotypes of a globally diverse population for four lodging resistance traits.

The globally diverse population of 795 genotypes was evaluated for four lodging resistance traits, viz., SM, M, PR, and cLr. The existence and extent of natural variation among these traits was estimated. A high level of divergence among genotypes and a strong correlation among the traits were observed for all studied traits. An identical and positively semi-skewed frequency distribution was observed for all traits in three populations (Figure 1; Table 1), which showed their suitability for the genome-wide association study. The statistical descriptive of studied traits were compared among the whole population, *japonica* subpopulation, and *indica* subpopulation. The ranges and average values of all studied traits for the whole population were similar to *japonica* subpopulation, while their ranges in *indica* subpopulation were shorter (Table 1). The average value of SM, M, and PR was higher in *japonica* subpopulation than that of *indica*, which may indicate the greater strength of the available *japonica* germplasm. A significant variation among the genotypes of the natural population was observed by two-way analysis of variance (ANOVA) and coefficient of variation (Table 1, Table 2). The genotypes showed a variable phenotypic response in various environments (years and blocks). There was no variation in SM among various years, and there was no interaction of genotypes with annual ecological and metrological conditions (Table 2). The genotypes showed a significantly variable phenotypic response in different blocks except for PR and M, while there was a significant interaction of blocks with genotypes. The significantly high values (0.87–0.97) of the repeatability parameter (*r* ^*2*^) along with broad sense heritability (*h* ^*2*^) demonstrated the genotypes as the main source of variation for lodging resistance, which was consistent in all three populations. The heritability in SM and cLr was relatively low, whereas the genotype effect (Table 1) as the main cause of variation was confirmed by the higher repeatability estimate.

**TABLE 1:** Statistical descriptive of four culm lodging resistance–related traits in whole population and *japonica* and *indica* subpopulations.

		Range	Mean ± SD	CV (%)	*h* ^*2*^ (%)	Vg	V(G x E)	Ve	Skewness	Kurtosis	*r* ^2^
SM (mm^3^)	Whole	0.13–23.35	6.23 ± 3.65	58.69	23.10	135.85	11.16	5.88	1.24	2.12	0.88
	Jap	0.13–23.35	6.64 ± 4.13	62.17	15.42	46.49	10.49	4.91	1.31	2.11	0.88
	Ind	0.44–21.67	6.02 ± 3.37	56.06	15.24	54.31	10.91	6.32	1.09	1.48	0.89
M (g.cm)	Whole	273.33–2306.67	824.9 ± 215.11	30.53	84.32	251,015.31	15,572.71	2853.91	0.65	1.36	0.97
	Jap	313.33–2306.67	844.35 ± 261.07	30.92	93.7	228,633.5	13,088.87	2694.50	1.16	3.64	0.97
	Ind	273.33–1650	815.08 ± 246.77	30.28	87.95	262,527.1	16,770.43	2912.38	0.33	−0.21	0.97
PR (g.cm^2^)	Whole	13.67–115.33	41.25 ± 12.59	30.53	55.55	912.22	261.21	54.17	0.64	1.37	0.87
	Jap	15.67–115.33	42.22 ± 13.05	30.92	73.07	881.47	281.12	47.38	1.16	3.63	0.86
	Ind	13.67–82.50	40.75 ± 12.34	30.28	47.59	913.64	248.83	57.69	0.33	−0.21	0.87
cLr (g/cm)	Whole	0.15–0.87	0.43 ± 0.14	33.19	24.46	0.12	0.03	0.01	0.51	−0.31	0.89
	Jap	0.17–0.82	0.42 ± 0.14	32.97	67.08	0.13	0.03	0.19	0.57	−0.28	0.89
	Ind	0.15–0.78	0.44 ± 0.15	33.27	24.72	0.12	0.03	0.50	0.47	−0.32	0.89

SD, standard deviation from mean; CV, coefficient of variation; h^2^, broad sense heritability; *r* ^2^, repeatability; V_g_, genotypic variance; V (G x E), genotypes to environment interaction variance; V_e_, environmental variance.

**TABLE 2:** Analysis of variance among the 795 genotypes of globally diverse population for lodging resistance traits.

Trait	SOV	DF	MS	Significance
SM	Genotypes (G)	794	51.524	***
	Years (Y)	2	15.488	NS
	G x Y	794	5.935	NS
	Blocks (B)	1	529.751	***
	G x B	794	10.756	***
PR	Genotypes (G)	794	901.352	***
	Years (Y)	2	57.641	***
	G x Y	794	57.461	NS
	Blocks (B)	1	330.984	NS
	G x B	794	260.496	***
M	Genotypes (G)	794	250,486.96	***
	Years (Y)	2	139,786.52	***
	G x Y	794	3283.60	NS
	Blocks (B)	1	19,437.789	NS
	G x B	794	15,453.52	***
cLr	Genotypes (G)	794	0.125	***
	Years (Y)	2	0.190	***
	G x Y	794	0.006	NS
	Blocks (B)	1	0.337	**
	G x B	794	0.030	***

SOV, source of variation; DF, degree of freedom; MS, mean square; *** significance, *p* > 0.001; **, *p* > 0.01; NS, *p* < 0.05.

The studied traits showed a significant positive correlation among them. However, the correlation between PR and cLr was nonsignificant (Table 3). The highest correlation was between PR and M. On the other hand, M and PR showed relatively less correlation with cLr (0.266 and 0.023) and SM (0.293 and 0.608), respectively. A similar correlation pattern was observed among traits in *indica* subpopulation, while in *japonica* subpopulation, the cLr was observed to be highly correlated with PR and M (0.799).

**TABLE 3:** Pearson correlation coefficient (r) calculated between 10 pairs of lodging-related traits in structured rice population of 795 accessions.

	PR	SM	M	cLr
PR	1	0.339	0.90	0.799
SM	0.293	1	0.339	0.68
M	0.894	0.608	1	0.799
cLr	0.023^ns^	0.740	0.266	1

Ns, non-significant at *p* < 0.05; other all values are significant at *p* < 0.05; below, diagonal are for whole population, while above diagonal are for *japonica* subpopulation.

### 3.2 Population structure and relative kinship

A genetically, morphologically, and geographically diverse sample of a large population was used in this study. The whole population was genotyped and resulted in 3.3 million SNPs after cleaning and filter screening. These SNPs covered almost 373,245,519 bp of the whole rice genome with an average SNP density of 12.3 SNPs per kb. The 3.3 million SNPs from the whole population of 795 genotypes were evaluated to estimate the kinship matrix and principal components (PCs) and resulted in a strong population structure. The PC1 revealed the maximum variation, which divided the whole population into two distinct groups. The same results were supported by a phylogenetic tree and a kinship matrix (Supplementary Figure S1). The group-I was composed of 289 *japonica* accessions, while the group-II framed the 506 *indica* genotypes, hence denoted as *japonica* and *indica* subpopulations, respectively. The other structural features were consistent with those previously reported (Zhang et al., 2009; Zhang et al., 2011).

### 3.3 Association mapping and QTL analysis

**FIGURE 2:**
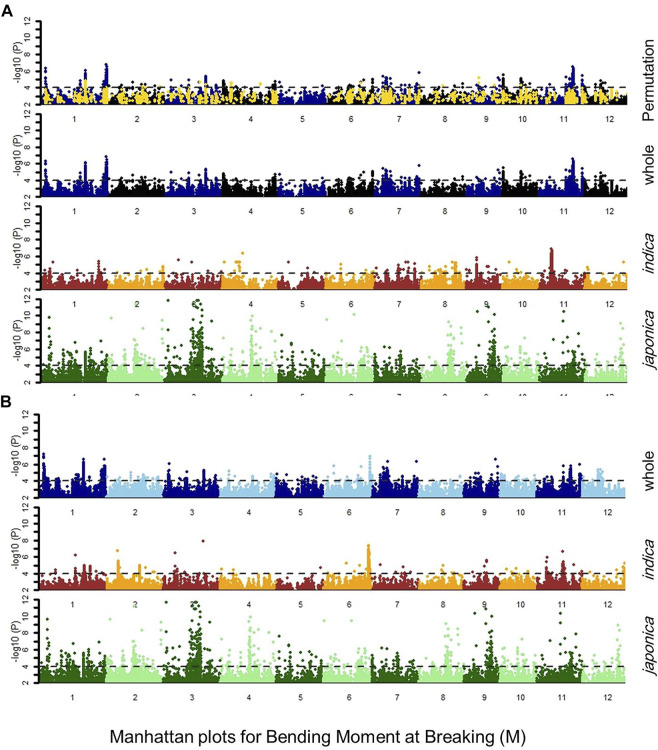
Manhattan plot for bending moment at breaking M, indicating the regression values for SNP markers in *japonica, indica* subpopulations, whole population, and whole population after 1000-times permutation with the **(A)** general linear model (GLM) and **(B)** compressed mixed linear model (cMLM), the dotted lines showed the threshold level at -log(P) ≥ 4.

The GWAS was conducted to detect the genomic regions associated with lodging resistance in rice. Depending upon population structure, GLM and cMLM regression analysis were adopted. In the first step, the association of SNP markers to lodging resistance traits was estimated by GLM and cMLM models for *japonica, indica* subpopulations, and the whole population separately (Figure 2, Supplementary Figures S2-S4). Then, the 1,000-time permutation with randomized phenotypes was performed by MLM to validate the GWAS results and to remove the false positives. The threshold–log10 (p) ≥ 4 was set for calling the significant associations. It is suggested that more association signals in a common region are caused by linkage disequilibrium (LD) decay, while the lower LD decay rate can reduce the power of association analysis for complex traits. The 0.28 and 0.25 LD decay rate with the 167 kb and 123 kb genome-wide *R* ^2^ reduction for *japonica* and *indica* subpopulations has already been reported (Huang et al., 2010; Huang et al., 2012). Hence, in this study, the genomic region containing a minimum of three significant SNPs within a 170 kb range was defined as a QTL. By these criteria, a total of 55, 113, 140, and 67 QTLs were observed for SM, PR, M, and cLr, respectively, which were defined by 2210, 766, 3548, and 383 significant SNPs (Table 4, and Supplementary Table S2). Among these QTLs, 15, 80, 70, and 11 QTLs were commonly observed in various populations and statistical models (Table 4).

**TABLE 4:** Summary of QTLs identified for lodging resistance traits in three populations by different regression models.

Traits	Whole population		*Indica* sub‐population		*Japonica* sub‐population		Common QTLs	Total QTLs
	GLM	MLM	GLM	MLM	GLM	MLM
SM	19	32	8	10	4	6	15	55
PR	13	19	3	9	73	82	80	113
M	18	45	13	18	64	63	70	140
cLr	17	21	8	23	6	7	11	67
Total	67	117	32	60	147	158	176	**375**

### 3.4 Biological correlation and pleiotropic QTLs

For the studied traits, some QTL regions were commonly detected for multiple phenotypes, which indicated the biological correlation among lodging resistance parameters. Among all of the identified QTLs, 33 QTL regions were found to be overlapped for more than one trait (Table 5). The QTL regions may be considered the potential pleiotropic loci for lodging resistance–related traits. The PR and M were observed as the most associated traits revealed by Pearson coefficient of correlation (Table 3, Table 5) and showed the maximum biological correlation with 20 overlapping QTLs. The one-third (Chigira et al., 2020) of total 33 QTLs were pleiotropically governing at least three lodging resistance traits. The maximum number of pleiotropic QTLs was found on chromosome 1, followed by chromosomes 7 and 11 with five and four QTLs, respectively. There was one genomic region of 316 kb on chromosome 7 (*qSM7-1*, *qPR7-1*, *qM7-2*, *qcLr7-1*), that was significantly associated with all four lodging related traits.

**TABLE 5:** List of QTLs with pleiotropic effect, situating at common QTL regions in the rice genome for lodging resistance traits.

Pleiotropic QTL	Chromosome	Start position	End position	Traits				QTLs count	Size of QTL (bp)	Candidate ORFs
				SM	PR	M	cLr			
1	1	3,538,854	3,683,583	qSM1-1	qPR1-3	qM1-1		3	144,729	19
2	1	5,566,150	5,744,781		qPR1-4	qM1-3	qcLr1-1	3	178,631	19
3	1	5,849,743	5,971,020	qSM1-3		qM1-4		2	121,277	14
4	1	6,271,684	6,376,084	qSM1-4	qPR1-5			2	104,400	17
5	1	6,581,990	7,070,411		qPR1-6	qM1-5		2	488,421	70
6	1	14,353,502	14,890,707		qPR1-7	qM1-7		2	537,205	73
7	1	24,957,693	27,134,674	qSM1-8		qM1-14, qM1-15	qcLr1-5	4	2,176,981	317
8	1	31,504,595	31,969,329		qPR1-13	qM1-19	qcLr1-6	3	464,734	74
9	1	32,366,242	33,807,002		qPR1-14	qM1-21	qcLr1-7	3	1,440,760	62
10	1	40,397,326	42,294,223	qSM1-12	qPR1-15	qM1-26		3	1,896,897	291
11	2	15,454,980	17,571,479		qPR2-5	qM2-4		2	2,116,499	311
12	2	24,657,034	24,799,865		qPR2-10		qcLr2-5	2	142,831	46
13	3	14,879,518	15,135,921	qSM3-2	qPR3-2	qM3-6		3	256,403	44
14	3	20,883,680	24,068,489		qPR3-10	qM3-13		2	3,184,809	479
15	3	27,631,508	27,677,782		qPR3-11		qcLr3-6	2	46,274	6
16	4	33,314,165	33,457,154		qPR4-12	qM4-12		2	142,989	24
17	5	5,161,253	5,348,092	qSM5-2			qcLr5-2	2	186,839	26
18	6	29,687,352	29,709,960	qSM6-2		qM6-6	qcLr6-3	3	22,608	3
19	7	5,261,202	5,577,829	qSM7-1	qPR7-1	qM7-2	qcLr7-1	4	316,627	52
20	7	7,234,758	7,608,752	qSM7-2		qM7-3		2	373,994	56
21	7	9,435,313	9,925,095	qSM7-3		qM7-4		2	489,782	64
22	7	10,111,738	11,910,229	qSM7-4		qM7-5		2	1,798,491	280
23	7	16,028,149	16,517,487		qPR7-3	qM7-7	qcLr7-3	3	489,338	74
24	8	18,109,039	18,657,995		qPR8-5	qM8-5		2	548,956	74
25	8	18,278,186	20,416,330	qSM8-1	qPR8-5	qM8-6		3	2,138,144	249
26	9	16,303,469	16,907,874		qPR9-4	qM9-8		2	604,405	96
27	10	871,334	1,056,808	qSM10-1		qM10-1		2	185,474	29
28	10	2,641,123	2,829,097	qSM10-2		qM10-2		2	187,974	30
29	11	7,873,690	8,601,084	qSM11-3, qSM11-4		qM11-2		2	727,394	110
30	11	15,327,195	15,530,521		qPR11-8	qM11-3		2	203,326	22
31	11	16,648,356	22,483,866	qSM11-5, qSM11-6	qPR11-9, qPR11-10	qM11-5	qcLr11-3, qcLr11-4, qcLr11-5, qcLr11-6	8	5,835,510	823
32	11	21,216,213	23,048,708	qSM11-8	qPR11-11	qM11-8		3	1,832,495	83
33	12	10,232,335	10,956,543	qSM12-2		qM12-1	qcLr12-1	3	724,208	94

### 3.5 Candidate gene identification

To evaluate the available candidate loci in identified QTLs, the SNPs in the physical range of annotated loci or their promoters were observed. A total of 4,031 annotated loci in a total of 30.1 Mb of the genomic region (Table 5) were revealed for pleiotropic loci in promoter and coding sequence (CDS) regions (Supplementary Table S3). The commonly identified QTL on chromosome 7 possesses 59 candidate genes. As per functional annotations, three out of a total of 59 genes, viz., *OsFBA2*, *OsFBX222*, and LTPL84-protease inhibitor may be considered the candidate loci for LR-related traits (Supplementary Table S3).

### 3.6 Functional annotations of candidate genes

**FIGURE 3:**
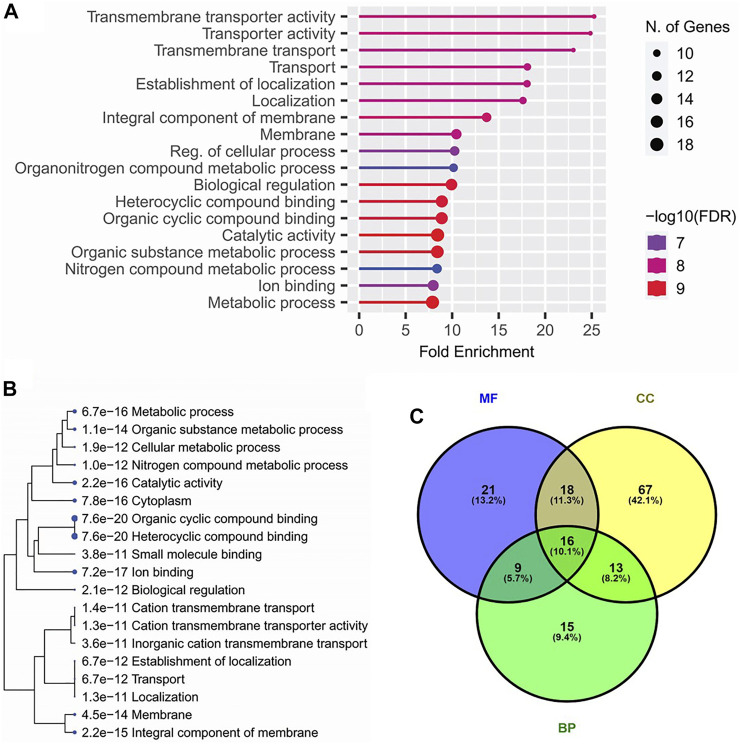
Top enriched gene ontological (GO) terms **(A)**, top enriched KEGG pathways **(B)** annotated for the candidate genes within the genomic regions of 33 pleiotropic QTLs detected for four lodging resistance traits, and Venn diagram **(C)** indicating the overlapping genes among GO terms including biological processes (BP), cellular components (CC), and molecular functions (MF).

All of the candidate loci for pleiotropic QTLs were subjected to finding the functional annotations. Among the various biological process-related gene ontological (GO) terms, ‘Transmembrane transporter activity’ and ‘Localization’ were observed as the top enriched GO terms. Among the cellular components, ‘membrane’, and among molecular functions, ‘Biological regulation’ were the top enriched GO terms (Figures 3A,C). Similarly, among the KEGG pathways, ‘Ion binding,’ ‘catalytic activity,’ ‘membrane’, ‘localization,’ and ‘transportation’ related pathways were significantly enriched (Figure 3B).

### 3.7 *In silico* and *in vitro* (qRT-PCR based) expression validation of candidate loci

**FIGURE 4:**
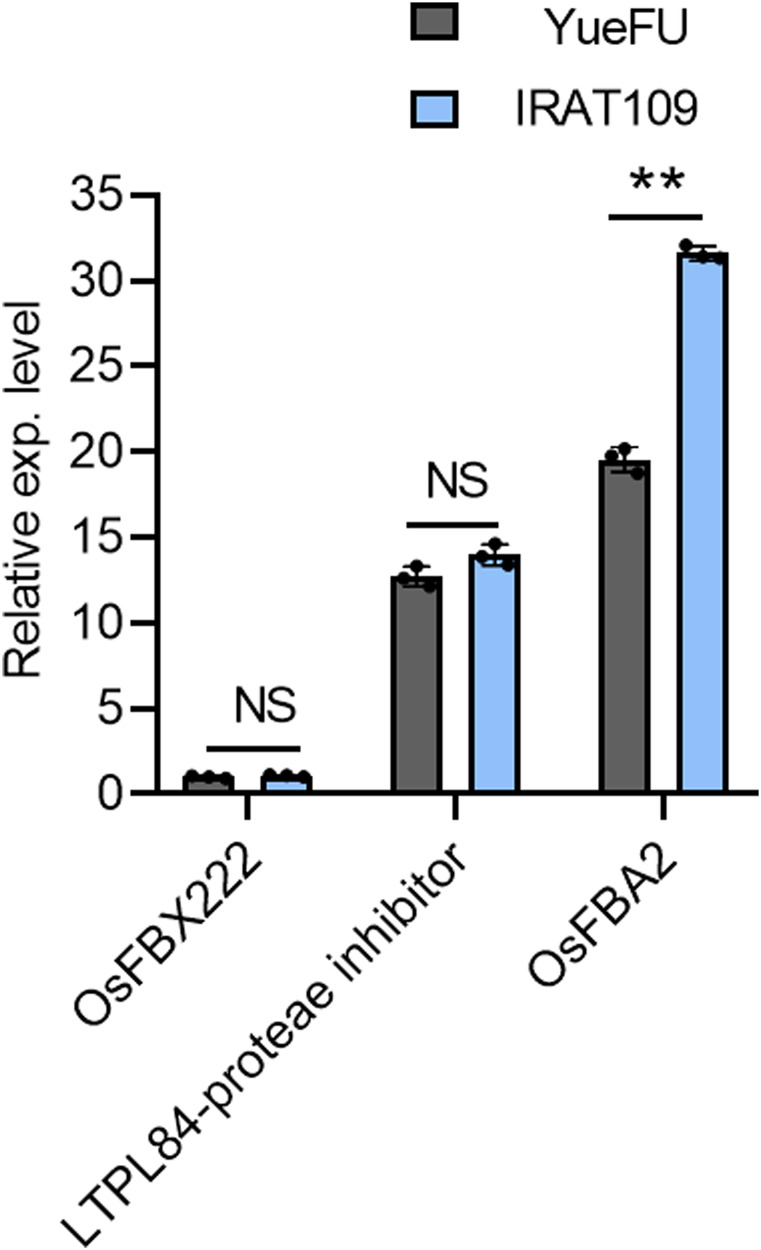
Gene expression analysis of three candidate genes in a pleiotropic QTL on chromosome 7 of the rice genome. NS; no significant difference, ** *p* < 0.01 based on two-tailed Student’s t-tests.

We further evaluated the candidate genes on chromosome 7 for their expression analysis. One strong and thick stem plant, IRAT109, and a weak and thin stem plant, YueFu, were used to analyze the expression of these three genes. The results showed a significant increase in expression levels of *OsFBA2* in strong plants, but the expression levels of *LTPL84*-proteae inhibitor and *OsFBX222* varied non-significantly. These results were consistent with the database of *in silico* expression analysis conducted on tissue-wise expression data extracted from the Ricexpro database (https://ricexpro.dna.affrc.go.jp/category-select.php). Moreover, *OsFBA2* has the most obvious difference in expression between the two germplasm (Figure 4). Hence, this gene may not only be considered a candidate for future studies on lodging resistance but also validated the authenticity of results from the current study.

### 3.8 Haplotype identification

**FIGURE 5:**
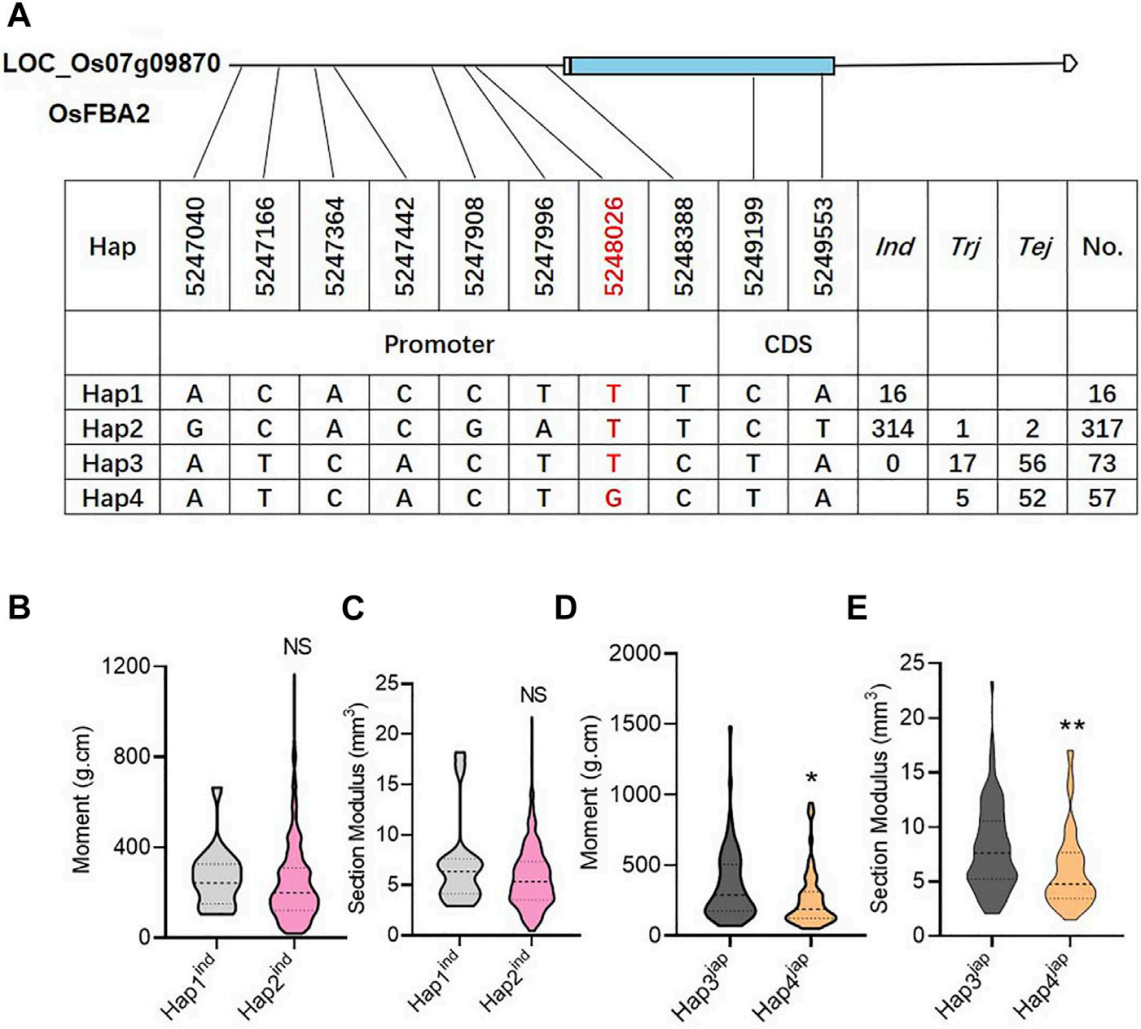
Haplotype identification of *OsFBA2*. **(A)** Candidate genomic region and haplotypes of *OsFBA2* from 463 rice cultivars, where *Tej*; temperate *japonica* population, *Trj*; tropical *japonica* population, *Ind*; *indica* population. **(B–E)** Phenotypic variation comparison among various haplotypes for bending moment at breaking **(B,D)**, section modules **(C,E)**. NS; no significant difference, * *p* < 0.05, ** *p* < 0.01 based on two-tailed Student’s t-tests.

A total of 463 genotypes and 10 SNPs (two SNPs in CDS and eight SNPs in promoter regions) were used to find the possible haplotypes of candidate genes. Consequently, the total gene pool could be classified with the availability of four haplotypes. Among these four haplotypes, Hap1 and Hap2 represented the *indica* sub-population, while Hap3 and Hap4 were mainly distributed in the *japonica* sub-population (Figure 5A). Among the four haplotypes, Hap3 was prominent, with significantly high values of M and SM (Figures 5B–D). Hence, the SNP marker at 5248026 bp physical position on chromosome 7 was considered a functional SNP to induce lodging resistance (Figure 5).

## 4 Discussion

Lodging is one of the major abiotic stress factors affecting yield loss of rice, especially in developing Asian countries (Khush, 1997). Historically, the direct and fundamental method to improve the breeding material for complex quantitative traits was morphological marker-assisted selection, where the frequency of favorable alleles could be increased within a specific population over multiple cycles of selection. Since the pre-green revolution, many breeding efforts have been made to improve lodging resistance with improved yield in rice (Meng et al., 2021). Nonetheless, the lodging resistance in rice crop showed negative correlation to some of the yield parameters as large spike for high yield made the genotypes prone to lodging (Hirano et al., 2017), dwarfism limits the total yield potential (Islam et al., 2007; Rana et al., 2021), and high fertilizer application with dense planting made the genotype vulnerable to lodging in windy and rainy seasons (Shah et al., 2019). Hence, the identification of potential QTLs and/or genes associated with lodging resistance traits and their manipulation in breeding material is the best way to achieve sustainable improvement. It has been reported that the culm strength for lodging resistance could be contributed by stem diameter (SD), stalk bending strength (SBS), and the pushing resistance in field conditions (Ishimaru et al., 2008; Hu et al., 2013), as a significant negative correlation of culm lodging rate with these three traits has been observed. Understanding the genetic basis of these traits will help to induce lodging resistance in the breeding gene pool.

In this study, a geographically and genetically diverse population of 795 rice genotypes was used to uncover the genetic architecture of culm lodging resistance in rice. The genotype heterogeneity within a genotype was removed, and purity was confirmed for 3 years by the SSD approach, and then phenotypes were investigated for 3 years. The wide-range, optimum normal frequency distribution and significant phenotypic diversity in the population for studied traits showed the polygenic control of traits. A significant genetic potential of genotypes for lodging resistance was observed by high heritability values. The high-resolution genotyping with 13X sequencing and 12.3 SNPs per kb markers density enhanced the power of genome-wide association analysis by improving mapping resolution and imputation efficiency (Huang et al., 2010). Hence the deep sequence data from a significant number of landraces acquired the maximum genetic diversity, which was a key target to reveal in GWAS (Rashid et al., 2022).

A genome-wide association study is an effective tool to dissect the genetic basis of complex traits like lodging resistance and its components. However, it is always pretentious due to the occurrence of false positives and true negatives (Atwell et al., 2010). In different studies on human and plant genomes, two factors, as estimation of population structure and genetic control, were observed to be suitable to avoid the detection of false-positive signals (Devlin and Roeder, 1999; Marchini et al., 2004; Yu et al., 2006). In the current study, the whole population was divided into *indica* and *japonica* subpopulations on the basis of population structure, Q-matrix, kinship-matrix, and PCs. Then, the marker trait association was separately and recurrently evaluated for the whole population and *indica* and *japonica* subpopulations with multiple regression modals including GLM, MLM, and cMLM. This approach has previously been reported to reduce false positives (Yang et al., 2010; Yang et al., 2011). The detection of a clear population structure as in *indica* and *japonica* subpopulations, the use of multiple regression models for association estimation, and the 1000-time permutation test significantly reduced the detection of false positives. Furthermore, GWAS resolution can be judged by LD estimation (Flint-Garcia et al., 2003). The 167 kb and 123 kb LD decay rate in *japonica* and *indica* subpopulations has been reported in previous studies (Huang et al., 2010; Huang et al., 2012). Thus, in this study, the threshold criteria of at least three SNPs in a 170 kb LD block were followed for defining the QTLs to enhance the power of GWAS resolution. Eventually, we mapped the 375 genomic regions significantly associated with lodging resistance traits in rice. The recurrently identified genomic regions for different traits showed their significant association with lodging resistance. These regions may be candidates for further gene cloning and marker-assisted breeding studies.

Previous genetic research studies on lodging traits used the GWAS approach and successfully identified the major QTLs explaining 65.7% of the variance for maximum load to breaking moment and critical mass (Hu et al., 2013). The identification of *SCM1*, *SCM2*, *SCM3,* and *SCM4* governing culm strength endorsed the contribution of SM, M, PR, and cLr to lodging resistance (Ookawa et al., 2010). Hence, we looked for the pleiotropic loci for these traits and found 33 overlapping QTLs for more than one trait. A strong correlation among traits was also observed from the phenotypic data. To confirm the analysis, a pleiotropic QTL on chromosome 7 governing all four studied traits was evaluated for candidate genes. Among the 59 open-reading frames in this QTL range, three genes, including F-box domain protein genes *OsFBA2*, *OsFBX222*, and a LTPL84-proteae inhibitor genes, were identified. We further evaluate the qRT-PCR based *in vitro* expression of candidate genes in thick-culm and thin-culm plants. The results showed the significantly high expression levels of LTPL84-proteae inhibitor and *OsFBA2* in strong plants, which confirmed the role of these genes in culm strength and lodging resistance. The F-box domain protein genes like *SCM2* have already been known to contribute to the culm strength in rice. We further evaluate the qRT-PCR based *in vitro* expression of candidate genes in strong and weak genotypes. A significant reduction in the expression level in weak genotypes confirmed the role of these genes in culm strength and lodging resistance. Hence, it could be proposed as a candidate gene for further investigation. The identification of candidate genes and their expression-based validation proved the accuracy and reliability of analytical methodology in current research. The isolation and manipulation of candidate genes in pleiotropic loci may be beneficial to enhance the culm strength, leading to lodging resistance in rice. Furthermore, using the same approach for other pleiotropic and non-pleiotropic loci, multiple candidate genes could be extracted (Supplementary Table S3).

The GO analysis of candidate genes from all pleiotropic QTLs supported the results for lodging resistance as the gene ontological terms for biological processes ‘transmembrane transport activity’ and ‘localization’ in cellular component ‘membrane’ were the most enriched, while ‘biological regulation’ was the most enriched molecular function. Similar research studies as strigolactone signaling (Sang et al., 2014; Yano et al., 2015) and the lignin accumulation (Yoon et al., 2015) to enhance the culm strength have been reported. These results were reinforced by the identification of the ‘Biosynthesis of Cofactors,’ ‘Sphingolipid Metabolism,’ and ‘Pyrimidine Metabolism’ pathways, which had their role in cellular localization and required for cell proliferation (Siddiqui and Ceppi, 2020).

The pyramiding of the candidate genes with other genes of agronomic importance may enhance the overall yield and culm strength in rice. Nonetheless, due to the epistatic nature of some genes, it may be necessary to find the most favorable alleles of candidate genes. Hence, we performed the haplotype analysis to reveal the functional allele for the candidate gene *OsFBA2* and observed that Hap3 was the most promising haplotype in *japonica* sub population. These genes have not been reported to contribute to culm strength. Hence, we speculate that *OsFBA2* is the most likely candidate gene for pleiotropic QTL-19 on chromosome 7 (Table 5). Further studies such as gene cloning and functional analysis for these two and the other identified candidate genes in this study will be helpful in understanding their genetic mechanism to enhance culm strength.

## Data Availability

The original contributions presented in the study are publicly available. This data can be found here: PRJEB6180.
