# Supplementary figures and images for: Unveiling the genetic architecture for lodging resistance in rice (Oryza sativa. L) by genome-wide association analyses
Source: Front Genet. 2022 Sep 6;13:960007. doi: 10.3389/fgene.2022.960007 (PMC9486067; doi:10.3389/fgene.2022.960007)

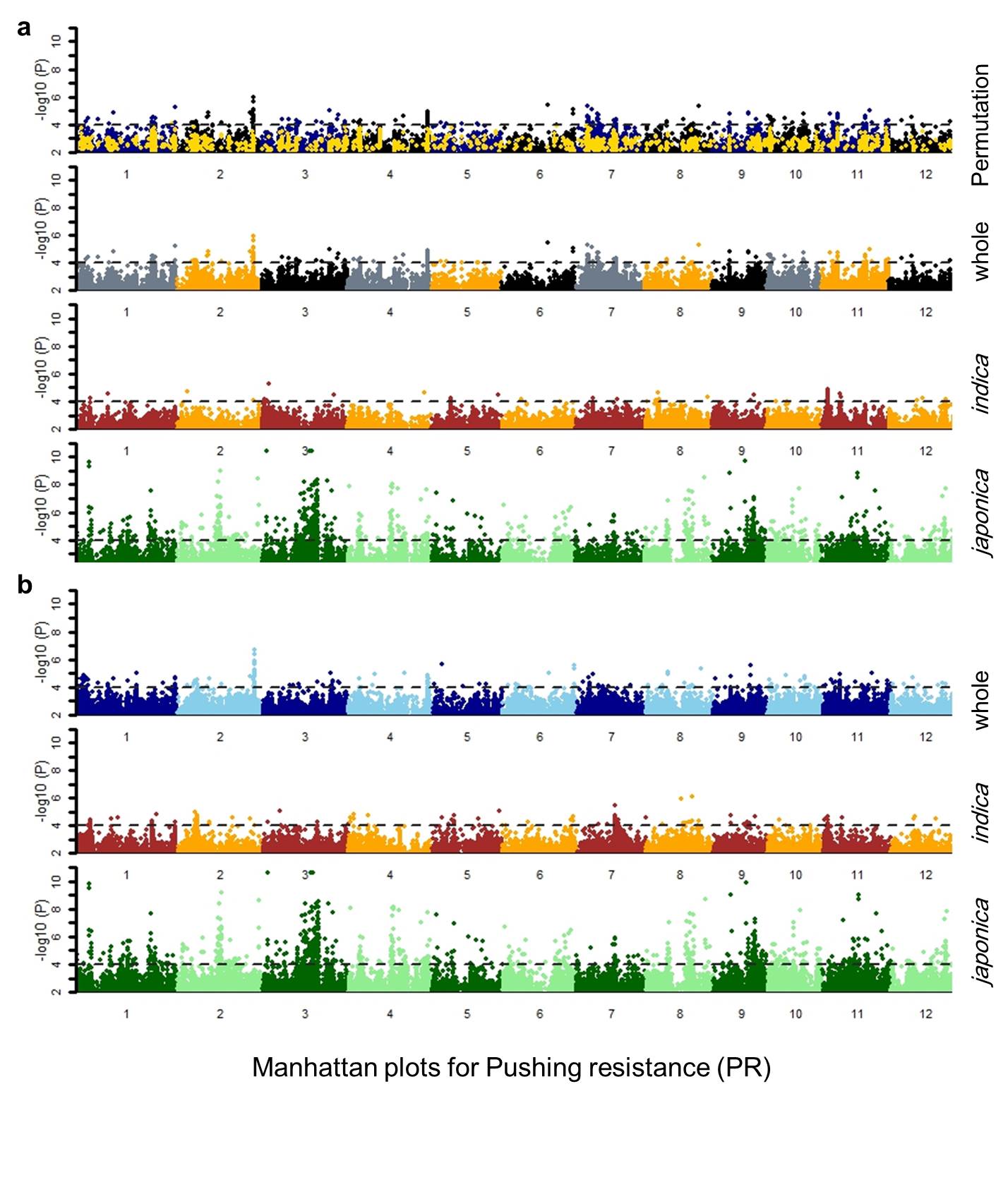

Supplement: Supplementary file 3 [file Image3.jpg]

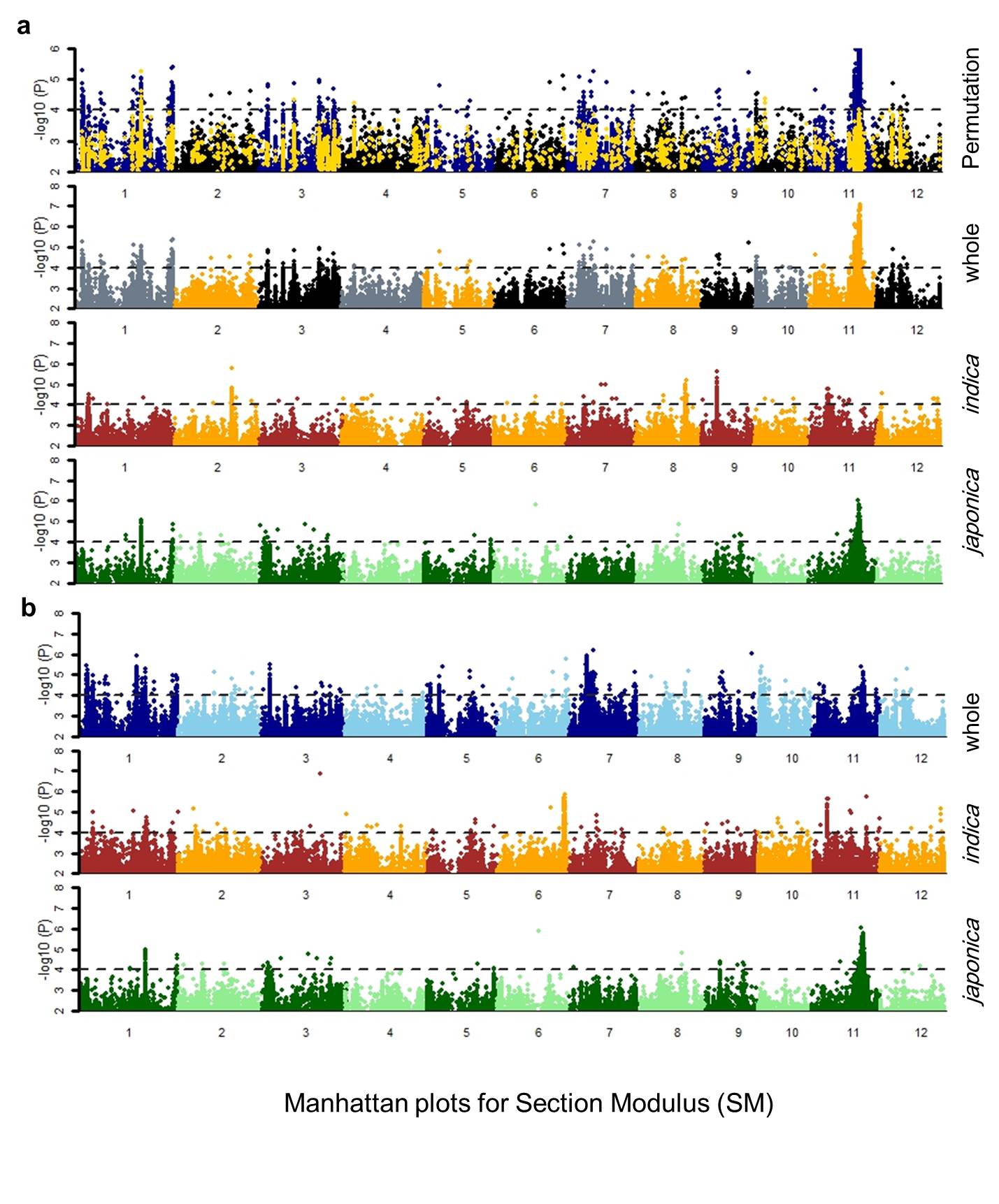

Supplement: Supplementary file 4 [file Image2.jpg]

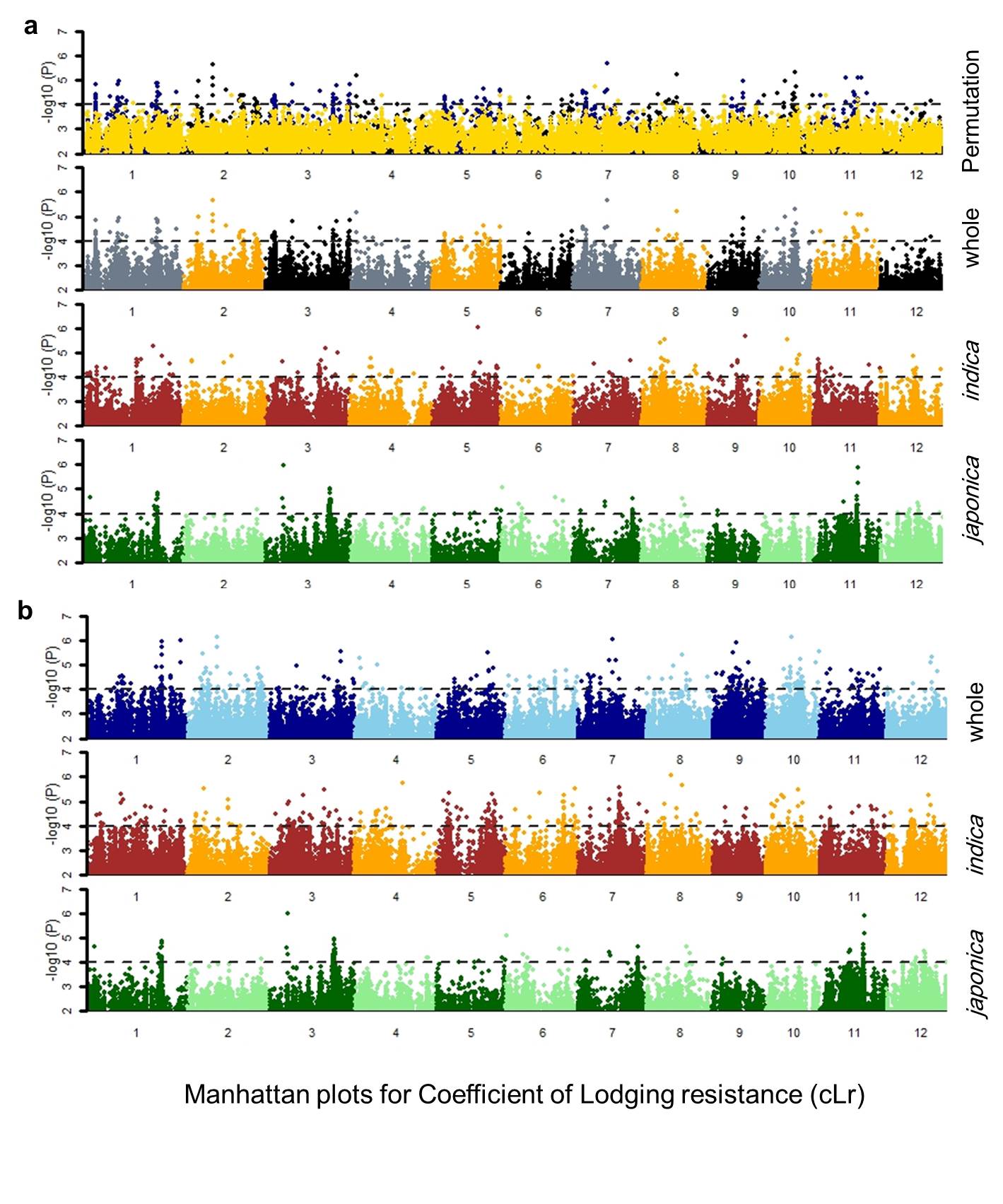

Supplement: Supplementary file 6 [file Image4.jpg]

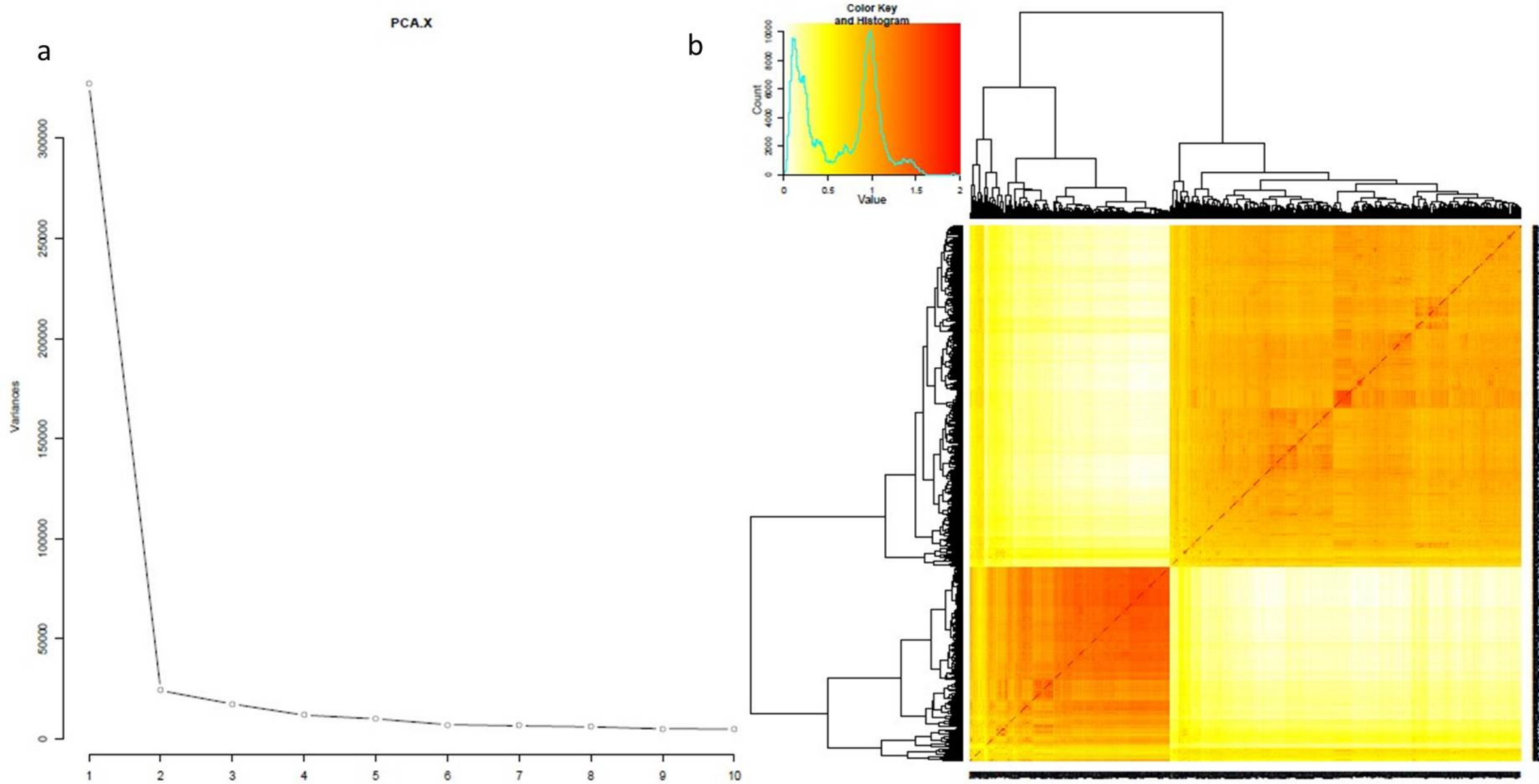

Supplement: Supplementary file 7 [file Image1.pdf]
